# Burden of morbidity in a patient perspective – the case of sick-leave certified patients in primary care

**DOI:** 10.1186/1471-2458-9-157

**Published:** 2009-05-27

**Authors:** Lennart Carlsson, Roland Morgell, Lars-Erik Strender, Britt Arrelöv, Gunnar H Nilsson

**Affiliations:** 1 Department of Neurobiology, Caring Sciences and Society, Center for Family and Community Medicine, Karolinska Institutet, Alfred Nobels allé 12, SE-141 83 Huddinge, Sweden; 2 County Council Administration, Unit of Social Insurance Medicine, Stockholm County Council, Box 17533, S-118 91 Stockholm, Sweden

## Background

### Epidemiology in terms of diseases or patients

Data on morbidity in Sweden are based on numbers of diagnoses, classified by the WHO system, the International Statistical Classification of Diseases and Related Health Problems (ICD) [1]. Registers of morbidity are administered by each county council. On national level registers are held by the National Board of Health and Welfare, but no diagnoses from primary care are so far collected. Statistics on patients being treated by various caregivers are available, but there are no possibilities to read out from the registers which patient had which diagnoses. Thus the epidemiologic knowledge is based on diseases and not on patients having the diseases.

Data on diagnoses, including primary care, are also found in statistics from the Swedish Social Insurance Administration (SIA), based on sick-leave certificates, to show the work disability situation in the local area, the region and on the national level.

So far, to our knowledge, no study has reported any comparison between these two types of registers. Furthermore, no efforts have been made to show the morbidity burden measured by groups of patients with combined diseases. Thus the burden of morbidity today is based on statistics of diseases an not on statistics of patients with more than one disease at each time; i.e. the complex health status of certain patients in terms of their co-/multimorbidity have so far not been the focus of much investigation. [2]

The research question of interest was to study the possibilities to describe the burden of morbidity in a population based on the combined data on diagnoses at the patient level.

### The Swedish sickness insurance system

During the past two decades interest in the complex phenomena of disease, sickness and impaired work capacity has increased in Sweden. [3-6] The sickness insurance system in Sweden covers the part of the population that is aged 16 to 64 years. Sickness absence must be sanctioned by medical certification, and the right to sick-leave cash benefits for individual patients is decided by clerks at the social insurance offices, based upon the information in a certificate issued by a physician.

### Patient medical record system

Documentation in the patient record regarding the patient's health problem, along with actions considered and taken, is required by Swedish law. The coding most often complies with the tenth revision of the ICD (ICD-10), issued along with a short primary health care (PHC) version "KSH97P" based on the full ICD-10 coding system [7]. In several county councils in Sweden including Stockholm County it is mandatory for at least one diagnosis to be registered for every contact between the patient and the physician. Depending on the type and version of the medical record being utilized, the additional health problems of patients with more than one defined diagnosis might be described in a free-text part of the medical record. Thus the diseases and health related problems registered in the diagnosis field in the medical record tend to be only part of the whole health problem spectrum, sometimes more fully described in the free-text part of the record [8].

### Aim of the study

The main aim of the study was to explore the burden of morbidity in a sick-leave registered population within primary care in a diagnosis and a patient perspective, based on diagnostic information from sick-leave certificates and patient medical records, respectively.

## Methods

### Data collection

During four weeks, spread out over the year to avoid periods including Swedish big holidays, every sick-leave certificate that was registered at one social insurance office in an area within the Stockholm County was collected. The first two weeks were in July 2004, the third week in February 2005 and the fourth week was in May 2005. In total 694 sick-leave certificates representing 578 patients were collected. About half of those were issued by primary care physicians, and thus 316 certificates representing 279 patients were used in this study. The information about each patient included encrypted identity number, age, sex and diagnoses registered, in a few cases more than one diagnosis per certificate.

For those 279 patients copies of their medical record were retrieved from their PHC centre, covering a period of twelve months before the date of issue of their certificate. The information about each patient included encrypted identity number, age, sex and diagnoses originally registered in the diagnosis field in the medical record. A secondary coding was then performed based on the information given about the patient's health status in the free-text area of the medical record. This secondary coding was carried out by a primary care physician involved in this study (Morgell), using guidelines for classification and coding in primary care, issued by Stockholm County Council [9].

Ethical approval has been applied for, and has been given for this study before data retrieval was made. (The Karolinska Institute Ethics Committee, Dnr 2004/5:9; today 'The regional board of ethical vetting in Stockholm')

### Data analysis in a diagnosis and a patient perspective

All diagnosis data were first analysed in a diagnosis perspective by comparing all diagnoses retrieved from the two different sources on a diagnosis group level according to the ICD-10 chapters.

Then same data were analysed in a patient perspective by applying the Adjusted Clinical Groups^® ^case-mix system (ACG), version 7.1, showing numbers of patients with combined diagnoses and their distribution in terms of patient categories – the ACGs. [10]

### Applying the Adjusted Clinical Groups^® ^system

The ACG system was developed at Johns Hopkins University in Baltimore, MD, USA [11,12]. This case-mix system is patient-oriented and patients are assigned to defined categories according to the condition of each individual. The original idea of the ACG system was to describe the health situation in a defined population based on the current health condition of each individual calculated according to the degree of risk of need for care in the future [11].

The case-mix system is labelling each one of the patients to fall into only one out of about 100 different patient categories (the ACGs), depending on each patient's combination of types of diseases. The types of morbidity have been classified by using five criteria simultaneously when defining every ICD-10 code to go into one of the 32 different groups of types of diseases, and those criterias were 1) the duration and 2) the severity of the condition, 3) the aetiology, 4) the diagnostic certainty and 5) the expected need for specialist care for the condition.

Thus the burden of morbidity in the population is based on the combined types of diseases of each patient. Thereby the distribution of ACGs shows a pattern of more or less complex co-/multimorbidity in the population.

## Results

### Background data

Medical records were retrieved from 279 patients in primary care; of those 33.3% were male and 66.6% female. The number of diagnoses retrieved from the sick-leave certificates amounted to 357 and the equivalent number of diagnoses in the medical records with diagnoses from the secondary coding added amounted to 1934.

### Main findings

It was feasible to monitor a pattern of morbidity burden in a defined population by building categories of patients based on each patient's combined types of morbidity. This pattern was different from building categories of diseases based on diagnoses.

### The diagnosis perspective

The comparison on diagnosis level between the two sources of data is shown in terms of ICD-10 chapters in Table 1. No big differences could be found in the comparison, although among the organ system oriented chapters the two most frequent chapters V ('Mental and behavioural disorders') and XIII ('Musculoskeletal system and connective tissue') together were more dominant in the sick-leave certificates – 82.8% versus 67.1%.

**Table 1:** The number of diagnoses in the 216 sick-leave certificates compared with the number of diagnoses in the 279 patient medical records, secondary coding of diagnoses included, sorted by ICD-10 chapters.

*Diagnosis* *Chapter* *(ICD-10)*		*# Diagnoses from sick-leave certificate* *(n = 357)*	*# Diagnoses from patient med. Record (n = 1934)*
V	Mental and behavioural disorders	127 (35.6%)	526 (27.3%)
XIII	Musculoskeletal system and connective tissue	121 (33.9%)	622 (32.3%)
XVIII	Symptoms, signs and abnormal clinical and laboratory findings, not elsewhere classified	27 (7.6%)	186 (9.7%)
X	Diseases of respiratory system	19 (5.3%)	120 (6.2%)
IX	Diseases of circulatory system	11 (3.1%)	99 (5.1%)
IV	Endocrine, nutritional and metabolic diseases	10 (2.8%)	92 (4.8%)
XXI	Factors influencing health status and contact with health services	9 (2.5%)	47 (2.4%)
XI	Diseases of the digestive system	9 (2.5%)	41 (2.1%)
XIX	Injury, poisoning and certain other consequences of external causes	5 (1.4%)	29 (1.5%)
VI	Diseases of the nervous system	3 (0.8%)	30 (1.6%)
Others	(nine chapters)	16 (4.5%)	135 (7.0%)

### The patient perspective

**Figure 1:**
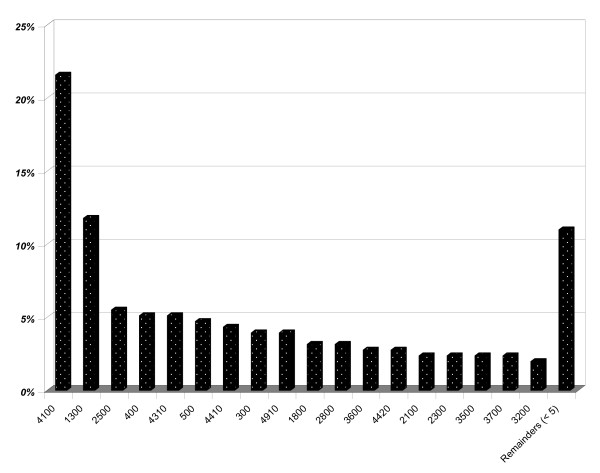
**The most frequent patient categories in terms of Adjusted Clinical Groups (ACGs) based on all diagnoses coded from the patient medical records, including secondary coding**. ACG # ACG Description. 4100 2–3 Other ADG Combinations, Age 35+. 1300 Psychosocial, w/o Psychosocial Unstable. 2500 Acute Minor/Psychosocial, w/o Psychosocial Unstable. 0400 Acute Major. 4310 4–5 Other ADG Combinations, Age 18 to 44, no Major ADGs. 0500 Likely to Recur, w/o Allergies. 4410 4–5 Other ADG Combinations, Age 45+, no Major ADGs. 0300 Acute Minor, Age 6+. 4910 6–9 Other ADG Combinations, Age 35+, 0–1 Major ADGs. 1800 Acute Minor/Acute Major. 2800 Acute Major/Likely to Recur. 3600 Acute Minor/Acute Major/Likely to Recur/Chronic Medical: Stable. 4420 4–5 Other ADG Combinations, Age 45+, 1 Major ADGs. 2100 Acute Minor/Likely to Recur, Age 6+, w/o Allergy. 2300 Acute Minor/Chronic Medical: Stable. 3500 Acute Minor/Likely to Recur/Psychosocial. 3700 Acute Minor/Acute Major/Likely to Recur/Psychosocial. 3200 Acute Minor/Acute Major/Likely to Recur, Age 12+, w/o Allergy. Remainders (< 5).

The pattern of morbidity resulting from the ACG grouping, based on the patient medical records, is shown in Figure 1. About ninety percent of all patients were represented in 18 different ACGs out of a total of 83 possible groups. About seventy percent of all patients belonged to ACGs that had a combination of two or more different types of morbidity. That means that some of the patients with a psychosocial classified diagnosis had a combination with other categories of diagnosis and thus were represented in the more complex groups ACG #4310, #4410 and #4910 besides the more homogeneous groups #1300 and #2500 with diagnoses only from the ICD-10 chapter V.

**Figure 2:**
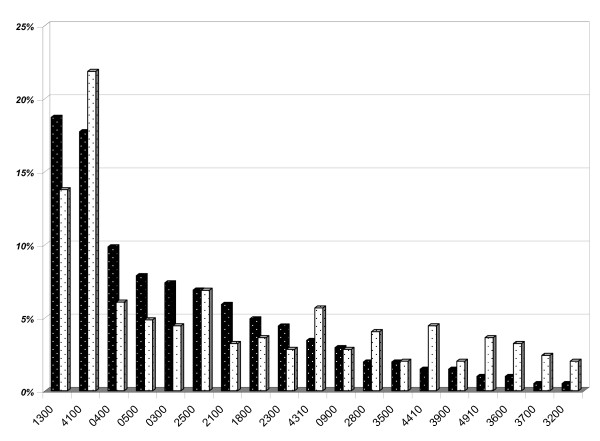
**Comparison between Adjusted Clinical Groups (ACGs) based only on originally recorded diagnoses (dark staples), and ACGs with all secondary coded diagnoses included (light staples) in the patient medical records**. (Top twelve in descending order by the originally recorded diagnoses; n = 279). ACG # ACG description. 1300 Psychosocial, w/o Psychosocial Unstable. 4100 2–3 Other ADG Combinations, Age 35+. 0400 Acute Major. 0500 Likely to Recur, w/o Allergies. 0300 Acute Minor, Age 6+. 2500 Acute Minor/Psychosocial, w/o Psychosocial Unstable. 2100 Acute Minor/Likely to Recur, Age 6+, w/o Allergy. 1800 Acute Minor/Acute Major. 2300 Acute Minor/Chronic Medical: Stable. 4310 4–5 Other ADG Combinations, Age 18 to 44, no Major ADGs. 0900 Chronic Medical: Stable. 2800 Acute Major/Likely to Recur.

An analysis was carried out stressing the difference between the originally registered diagnoses in the medical record and the results of the secondary coding from the free-text part of the record added. An ACG grouping was performed to compare the difference in the morbidity pattern influenced by the secondary coding. The two ACG distributions are compared and shown in Figure 2, where an obvious shift to more complex groups of patients can be seen when adding more diagnoses to each patient. When grouped only by the originally registered diagnoses no more than just above 50% of all patients had a combination of two or more different types of morbidity, and the proportion rose to just above 70% when adding the secondary coded diagnoses.

## Discussion

### Limitations and strengths

The main limitation of the study is that a systematic sample of patients was used, despite the efforts to minimize the seasonal variations. Further, the data from the sick-leave certificates were captured only once in order to obtain the current reason for disability to work, while data from the medical records were retrieved from a twelve months' period prior to the issue of the certificate, in order to capture the actual health status of the patient included in the study. [13]

One of the strengths of the study is the work that has been carried out to add more diagnosis codes to those originally registered. However, the most valuable result is the possibility to show the individual profiles of combined morbidity types, constituting a pattern of morbidity burden in a population based on combined diagnoses from each patient. Thereby this study challenges the traditional way of studying epidemiological issues in health care, adding the patient perspective to the mere counting of diagnoses and diseases.

Different periods of the year have been chosen for data retrieval to reduce the impact of potential seasonal variations in the diagnosis' distribution. Our study is not intended to explore such impact, and our data are not sufficient to evaluate that aspect. When the previous diagnoses of each person are included they cover all times of the year.

### Main findings

The main finding in this study was that the pattern of morbidity burden in a defined population could be elucidated by building categories of patients based on each patient's combined types of morbidity instead of building groups of diagnoses in ICD-10 chapters.

### The diagnosis perspective

The diagnoses with musculoskeletal and mental behavioural problems were most frequent among all patients, but were more dominant when measured by the data in the certificates. When matching the data from the certificates with data from the originally registered diagnoses in the medical record some dissimilarity could be seen. Quite a few diagnoses in the certificate could not be found in the diagnosis field in the medical record from the actual patient, but could sometimes be understood from the information in the free-text part of the record.

It should not be surprising that the certificates have fewer – and other – diagnoses than what could be found in the medical record depending on the task of the two different registers to show either the working disability situation or the medical aspects of the health status.

### The patient perspective

The burden of morbidity in terms of patient categories, the ACGs, with combined data on diagnoses from each patient, elucidated another pattern of morbidity that might challenge the traditional epidemiological approach, based on statistics on diagnoses solely. [14-18]. Quite a few complex health states could be seen when patient categories were built; more than two thirds of all patients in the study showed a co-morbid status.

To our knowledge, no study has reported the individual burden of morbidity among sick-leave certified patients, nor have various sources of diagnosis been compared. The more complex pattern seen when using data from the medical records – including the secondary coding – is not surprising as more diagnoses are added at each patient.

However, the results of the analysis points out that some questions must be posed concerning how the information in the two types of sources should be used. [19,20]

Consequently, efforts are needed to ensure that more clinical information from the medical record is at hand when planning and implementing the caring, curing and rehabilitation processes. First and foremost, however, the patient perspective must be focused upon in order to provide a fair and solid basis for use in population based registers in epidemiological reports, and later in research and development as well as for educational purposes.

### Coding aspects

Greater attention must be directed towards the coding of diagnoses in medical records. During the secondary coding process quite a few diagnoses were found in the free-text part of the medical record. If used alone, the data from the coded diagnosis field in the patient record might not give a fair view of the health status of the patient. [21-23]

The reimbursement system for PHC in Stockholm County Council requires the physician to code the diagnoses that were the main reasons for consultation. The extent to which recording every diagnosis predisposes to medicalisation is unclear, but the ACG system minimizes the possibility by combining similar diagnoses into categories rather than counting them individually and adding them. Additionally, recording of any given diagnosis does not imply that any management strategies are instituted.

## Conclusion

The burden of morbidity for sick-leave certified patients in terms of ACG patient categories elucidated a pattern of the morbidity burden that must be taken into account when describing and measuring epidemiological aspects in primary care. Patient based grouping of data from medical records, including a secondary coding of free-text parts, reveals a more complex pattern of the morbidity burden than can be shown by compilation of diagnoses.

Efforts to improve the completeness of coding of diagnoses in medical records are urgently needed.

The results based on the patient perspective could challenge the traditional approach of epidemiology based solely on the statistics of diagnoses.

## Competing interests

The authors declare that they have no competing interests.

## Authors' contributions

LC performed the ACG work, analysed the data, and wrote the manuscript. RM contributed to the design and coordination of the study, shared the acquisition of data, and contributed to the writing of the manuscript. LES contributed to the design of the study, and drafted and examined the manuscript. BA and GN contributed to the design of the study, and the analysis of the data. GN also helped coordinating the study. All authors read and approved the final manuscript.

## Pre-publication history

The pre-publication history for this paper can be accessed here:


